# Assessment of the Presence of Free-Living Amoebae in Soil Samples from the Northwest Region of Spain Using Culture and Molecular Assays

**DOI:** 10.3390/microorganisms13051065

**Published:** 2025-05-02

**Authors:** Patricia Pérez-Pérez, Iván Rodríguez-Escolar, Elizabeth Córdoba-Lanús, Angélica Domínguez-de-Barros, Omar García-Pérez, José E. Piñero, Rodrigo Morchón, Jacob Lorenzo-Morales

**Affiliations:** 1 Instituto Universitario de Enfermedades Tropicales y Salud Pública de Canarias (IUETSPC), Universidad de La Laguna (ULL), 38206 San Cristóbal de La Laguna, Spain; pperezpe@ull.edu.es (P.P.-P.); acordoba@ull.edu.es (E.C.-L.); angelica4realejos@gmail.com (A.D.-d.-B.); ogarciap@ull.edu.es (O.G.-P.); jpinero@ull.edu.es (J.E.P.); 2 Departamento de Obstetricia y Ginecología, Pediatría, Medicina Preventiva y Salud Pública, Toxicología, Medicina Legal y Forense y Parasitología, Universidad de La Laguna, 38200 San Cristóbal de La Laguna, Spain; 3 Zoonotic Infections and One Health GIR, Laboratory of Parasitology, Faculty of Pharmacy, University of Salamanca, 37007 Salamanca, Spain; ivanrodriguez@usal.es (I.R.-E.); rmorgar@usal.es (R.M.); 4 Centre for Environmental Studies and Rural Dynamization (CEADIR), University of Salamanca, 37007 Salamanca, Spain; 5 Centro de Investigación Biomédica en Red de Enfermedades Infecciosas (CIBERINFEC), Instituto de Salud Carlos III, 28029 Madrid, Spain; 6 Biomedical Research Institute of Salamanca (IBSAL), University of Salamanca, 37007 Salamanca, Spain

**Keywords:** soil, *Acanthamoeba*, *Vermamoeba vermiformis*, seasonality, Castilla y León, Spain

## Abstract

Free-living amoebae (FLA) such as *Acanthamoeba* spp., *Balamuthia mandrillaris*, *Naegleria fowleri*, *Sappinia* spp., *Vahlkampfia* spp., and *Vermamoeba vermiformis* are naturally widespread in the environment, causing rare but fatal and debilitating infections in humans. In the present study, a total of 87 soil samples were collected from four provinces in the autonomous community of Castilla y León, Spain. These samples were collected in three different seasons during 2022–2023 (t1–t3) and were analysed by culture and molecular techniques (conventional PCR/sanger sequencing and qPCR). The obtained data revealed that the genus *Acanthamoeba* and the species *Vermamoeba vermiformis* were the most prevalent FLA. Furthermore, other genera/species of FLA were identified in the tested soil sources, suggesting a rich microbial biodiversity in Castilla y León soils. In addition, this study provides an important basis for future research on the ecology of these organisms and their potential impact on public health and the environment.

## 1. Introduction

Free-living amoebae (FLA) are aerobic, mitochondriate, eukaryotic protists widely distributed in nature, for example, in soils, freshwater, marine waters, and on the aerial parts of plants and animals. They are considered cosmopolitan in nature [1,2]. They include organisms from various supergroups such as Amoebozoa, Rhizaria, and Opisthokonta. Among the diverse FLA present in the environment, only a few genera and species, such as *Acanthamoeba* spp., *Balamuthia mandrillaris*, *Naegleria fowleri*, *Sappinia pedata*, *Vahlkampfia* spp., and *Vermamoeba vermiformis,* are pathogenic and opportunistic, capable of causing infections in humans and animals [3]. A number of *Acanthamoeba* species with various genotypes are responsible for the chronic illness known as granulomatous amoebic encephalitis (GAE), which mostly affects immunocompromised hosts, but it is also a causative agent of amoebic keratitis (AK) and skin lesions [4]. *Balamuthia mandrillaris* can also affect the central nervous system, causing *Balamuthia* amoebic encephalitis (BAE) in both immunocompromised and immunocompetent hosts, especially in very young or very old individuals. To a lesser extent, it can also cause epithelial lesions [3,5]. In contrast, children and young adults who have recently been in contact with warm freshwater are susceptible to an acute, fulminating, necrotizing infection of the central nervous system known as primary amoebic meningoencephalitis (PAM) caused by *Naegleria fowleri* [6]. *Sappinia pedata*, previously described as *S. diploidea*, also has caused a case of amoebic meningoencephalitis [7]. AK can also be caused by other pathogenic FLA, such as *Vermamoeba vermiformis* and vahlkampfiid amoebae, not exclusively by *Acanthamoeba* [8,9,10]. As a result, *V. vermiformis* has been identified to act as a reservoir of pathogens and as the etiological agent of keratitis, a painful ulcer close to the eye, and meningoencephalitis [11,12,13]. In general, the life cycle of FLA consists of two stages: a vegetative trophozoite stage, and when cells are exposed to extreme circumstances, they differentiate into double-walled cysts [14], except for certain FLA, such as *N. fowleri*, which presents a flagellated stage [3].

The FLA of clinical relevance feed on bacteria in the environment, but some of these bacteria manage to resist digestion and survive inside, transforming FLA into microbial reservoirs. This interaction not only reveals a bacterial survival strategy but also connects directly to the ecological role of environmental FLA as regulators of microbial communities and key players in nutrient recycling in ecosystems [15,16]. On the other hand, they are widely studied throughout the world because these FLA infections are classified as emerging diseases, given that the number of cases reported globally is rising annually and that the incidence of climate change indicates that it will inevitably continue to rise [17,18].

However, environmental studies on FLA epidemiology are scarce in Spain, and most of them have been carried out on *Acanthamoeba* on the Canary Islands [19,20,21,22] and the central area of Spain [23,24] by sampling water from different sources. In fact, there is much unknown about the presence of these protozoa in the soils in Spain. Therefore, the following study has been undertaken to investigate the distribution and abundance of FLA (pathogenic and non-pathogenic FLA) in soil samples from the North Central Iberian Peninsula (autonomous community of Castilla y León) during three different periods of the year. Considering the outcomes, the goal is to increase public awareness by highlighting the grave risk that the soil poses to public health.

## 2. Materials and Methods

### 2.1. Location and Sampling

The sampling area was in the autonomous community of Castilla y León, Iberian Peninsula, Spain, with an area of 94,224 km^2^, which is the largest region in Spain and one of the largest in Europe. It is made up of 9 provinces (León, Zamora, Salamanca, Valladolid, Palencia, Burgos, Soria, Segovia, and Ávila). Castilla y León’s climate is classified as Mediterranean according to Köppen, meaning that it experiences short, warm summers (average temperatures of 19 to 22 °C) and lengthy, cold winters (average temperatures in January of 3 to 6 °C). Rainfall during the summer is minimal, with an average annual precipitation of approximately 450–500 mm, which is more pronounced in mountainous areas. Furthermore, various sub-climates can be identified due to the territory’s considerable extension and orographic diversity. A large part falls within the temperate with a dry summer (Csb) or temperate with a dry season and temperate summer (Cfb) sub-climates, characterised by the average of the warmest month being below 22 °C but above 10 °C for ≥5 months. In several areas of the central plateau, the sub-climate is classified as temperate with dry or hot summer (Csa), as it exceeds 22 °C during the summer, or cold steppe (BSk), with average annual temperatures below 18 °C. At higher altitudes in mountainous regions, the climate transitions to cold temperate, marked by average temperatures below 3 °C during the coldest months and dry summers (Dsb or Dsc) [25].

**Figure 1:**
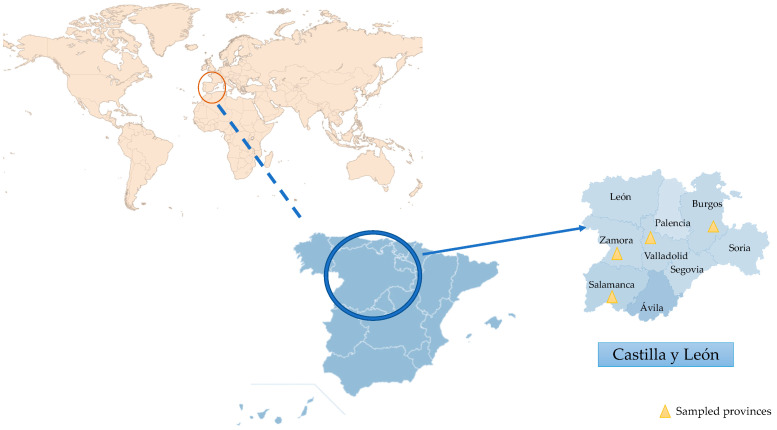
Geographical localization of the autonomous community of Castilla y León.

A total of 87 soil samples were collected from 29 sampling points across four provinces in Castilla y León: Salamanca (SS1–19), Valladolid (VS1–5), Zamora (ZS1–4), and Burgos (BS1) (Figure 1). Soil collection was carried out during three distinct periods, selected to reflect seasonal variation in environmental conditions: (1) September–November 2022 (t1, autumn), (2) January–February 2023 (t2, winter), and (3) May–June 2023 (t3, late spring). The same locations were sampled in each period to assess the temporal dynamics in the presence and diversity of these parasites. The evaluated samples represented a range of environments: parks (39/87), vegetable patches (18/87), riverbanks (9/87), pond edges (12/87), swamps (6/87), and beach sands (3/87). All samples were collected directly from the ground using sterile 15 mL tubes and stored at 4 °C until laboratory processing (Table 1).

### 2.2. Free-Living Amoebae Isolation

In total, 0.6 g of each soil sample was seeded directly onto 2% Non-Nutrient Agar (NNA) plates with a layer of heat-killed *E. coli*, forming a line, which were then incubated at 26 °C. The presence of amoebic cells was then assessed daily by monitoring the plates. Following the morphological characteristics using the Page key [26], plates with amoebae growth were cloned by dilution in NNA until a monoxenic culture was achieved, when possible.

### 2.3. DNA Extraction

DNA from positive samples was isolated from 1 to 2 millilitres of amoebic culture suspension for molecular analysis. To obtain the amoeba suspension, 4 mL of Page’s Amoeba Solution (PAS) was added to the plate with the monoxenic amoeba culture. Following the manufacturer’s instructions and the previously described method [27], the plate was scraped, the suspension was centrifuged, and the concentrated amoeba culture was directly placed into the Maxwell^®^ 16 tissue DNA purification kit sample cartridge (Promega, Madrid, Spain). Extracted DNA yield and purity were quantified using the NanoDrop Lite Spectrophotometer.

### 2.4. PCR and Molecular Characterizations

PCR amplification of the 18S rRNA gene from the extracted DNA was performed using universal primers: FLA-f 5′-CGCGGTAATTCCAGCTCCAATAGC-3′/FLA-r 5′-CAGGTTAAGGTCTCGTTCGTTAAC-3′ [28] and specific primers: JDP-1f 5′-GGCCCAGATCGTTTACCGTGAA-3′ and JDP-2r 3′-TCTCACAAGCTGCTAGGGAGTCA-5′ for amoeba presenting morphology corresponding to *Acanthamoeba* spp. [29] and Hv1227f 5′-TTACGAGGTCAGGACACTGT-3′ [30] /VermRV 5′-TGCCTCAAACTTCCATTCGC-3′ [31] for *Vermamoeba vermiformis*. For the family Vahlkampfiidae we used these primers: VAHL1 5′-GTCTTCGTAGGTGAACCTGC-3′ and VAHL2 3′-CCGCTTACTGATATGCTTAA-5′ [32].

Amplification reactions were performed with a total of 50 μL of mixture, containing 80 ng of DNA, and the PCRs were carried out in 40 cycles with denaturation (95 °C, 30 s), annealing (55 °C, 30 s) and primer extension (72 °C, 30 s) for FLA universal primers. Nevertheless, for *Acanthamoeba* spp. and *V. vermiformis* primers, the 50 μL PCR mixture contained 40 ng of DNA yield and 35 cycles with denaturation (95 °C, 30 s), annealing (50 °C, 30 s), and primer extension (72 °C, 30 s). For VAHL primers, amplification reactions were performed in a 50 μL mixture containing 60 ng of DNA, and the PCRs were performed in 35 cycles with denaturation (95 °C, 60 s), annealing (55 °C, 90 s) and primer extension (72 °C, 120 s). A primer extension of 7 min at 72 °C was maintained after the last cycle. Amplification products from all PCRs were analysed by electrophoresis through a 2% agarose gel and positive PCR products were sequenced using a Macrogen Spain service. Sequence homology analysis was used to identify several species by comparing them to DNA sequences found in the Genbank database from the National Library of Medicine (NCBI).

### 2.5. Multiplex Quantitative Real-Time PCR Assay (q-PCR)

A q-PCR reaction was performed on the samples that did not show amplification by conventional PCR, following the methodologies described by Córdoba Lanús et al. (2024) [31] and Qvarnstrom et al. (2006) [30].

The q-PCR reactions were performed in a final volume of 10 μL using 10 X TaqMan^®^ Multiplex Master Mix (Applied Biosystems, ThermoFisher Scientific, Waltham, MA, USA), 0.5 μM of each primer, 0.25 μM of probe, and 2 μL of DNA sample. This was set up on a QuantStudio 3 real-time PCR machine (ThermoFisher Scientific, Waltham, MA, USA) under the following conditions: a first step of 95 °C for 3 min, followed by 35 cycles consisting of two steps of 95 °C for 15 s and 60 °C for 1 min [30,31].

To confirm whether the *Acanthamoeba* spp. detected in the previous step corresponds to the T4 genotype, which accounts for more than 86% of *Acanthamoeba* keratitis worldwide [33], the ParoReal *Acanthamoeba* T4 kit (Ingenetix, GmbH, Vienna, Austria) was used. The qPCR reaction was performed on a QuantStudio 3 real-time PCR thermal cycler (ThermosFisher Scientific, MA, USA). This kit uses a FAM-labelled probe for the detection of a T4-positive genotype sample and a Cy5-labelled internal control.

### 2.6. Phylogenetic Analysis

The obtained sequences in this study were aligned using the software MAFFT (Version 7) with the accurate L-INS-i method [34] and trimAl for the removal of poorly aligned sites when necessary [35]. RAxML v.8.2.10 was used for phylogenetic analyses using the maximum likelihood algorithm [36]. RAxML used the GTRGAMMA substitution model for nucleotide alignments. The analysis was performed with 500 bootstrap replicates to assess the robustness of the branches. The tree was rooted with an external group.

## 3. Results

In this study, we monitored the environmental FLA community capable of growing at 26 °C in soil collected from four provinces of Castilla y León over the course of one year. From the total of 87 samples, all soil samples were positive for the presence of environmental FLA in NNA plates (87/87; 100%). After analysis of the 18S rRNA gene (the DF3 region in the case of *Acanthamoeba*), 74 soil samples (74/87; 85.05%) were positive for PCR. In addition, 7 samples (7/87; 8.04%) were q-PCR positive. All samples were found to be positive for *Acanthamoeba* spp. (Table S1).

*Acanthamoeba* spp. were the most abundantly isolated genus in soils throughout the years 2022 and 2023, with a total of 47 samples (47/87; 54.02%), with the T4 genotype being the most common (39/47; 82.98%). In contrast, the presence of *V. vermiformis* in 31.03% was outstanding (27/87) because it is the second most prevalent amoeba reported. Other amoebae belonging to the family Vahkampfiidae were also isolated and confirmed by conventional PCR/sanger sequencing, e.g., species of the genus *Naegleria* (*Naegleria australiensis*) or of the genus *Vahlkampfia* (*Vahlkampfia avara*).

The nucleotide sequences determined from this study were submitted to GenBank and assigned the following accession numbers: PP707062:PP707084, PP835514:PP835534, and PQ819023-PQ819044. All of them presented ˃ 95% of homology with the available DNA sequences in this database.

**Figure 2:**
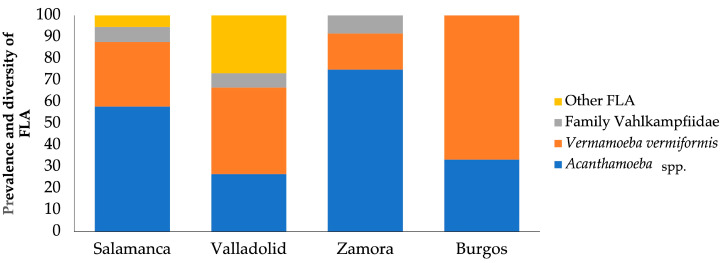
Distribution of free-living amoebae isolated in soil samples for each province.

The results obtained show that, regarding the provinces, out of the 87 samples collected, 57 originated from Salamanca, 15 from Valladolid, 12 from Zamora, and 3 from Burgos (Figure 2).

In Salamanca, a prevalence of 100% (57/57) was obtained, with the genus *Acanthamoeba* predominating with 57.90% (33/57). The species *V. vermiformis* was present in 29.82% (17/57) and amoebae of the family Vahlkampfiidae in 7.01% (4/57). However, other FLA were identified in 5.26% (3/57). Other FLA are those amoebae that are difficult to identify by a morphological approach.

In Valladolid, a prevalence of 100% (15/15) was obtained. However, *V. vermiformis* was identified in more samples with 40% (6/15). The genus *Acanthamoeba* was isolated in 26.67% (4/15), and from the family Vahlkampfiidae, only *N. australiensis* was observed in 6.67% (1/15). Other FLA were also found in 26.67% (4/15).

In Zamora, a prevalence of 100% (12/12) was obtained, with the genus *Acanthamoeba* being the most identified, with 75% (9/12). In addition, the species *V. vermiformis* was observed in 16.67% (2/12) and *Vahlkampfia* sp. in 8.33% (1/12).

In Burgos, a prevalence of 100% (3/3) was obtained with a predominance of the species *V. vermiformis* with 66.67% (2/3), while the genus *Acanthamoeba* was identified in 33.33% (1/3).

**Figure 3:**
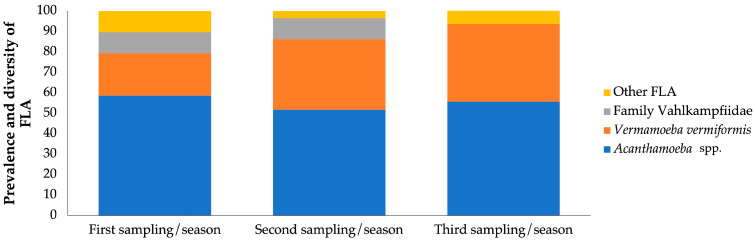
Distribution of free-living amoebae isolated in soil samples for each season.

According to the seasons, 29 samples were collected for each of the stations (t1–t3) out of the 87 samples that were distributed among the 29 sampling points (Figure 3).

In the first sampling/season (t1) a prevalence of 100% was obtained in all provinces, and a great diversity was observed with the genus *Acanthamoeba* in a large number of samples (17/29; 58.62%), the species *V. vermiformis* (6/29; 20.67%), and the amoebae of the family Vahlkampfiidae (3/29; 10.34%). Finally, other FLA were found (3/29; 10.34%).

In the second sampling/season (t2), a prevalence of 100% was obtained in all provinces. The different types of amoebae were observed, and among them, the genus *Acanthamoeba* (15/29; 51.72%;) and the species *V. vermiformis* (10/29; 34.48%) were the most abundant. In contrast, amoebae belonging to the family Vahlkampfiidae were present in 10.34% (3/29) and other FLA in a single sample (1/29; 3.44%).

In the third sampling/season (t3), a prevalence of 100% was obtained in all provinces. However, it showed a decrease in the diversity of pathogenic FLA with the genus *Acanthamoeba* (16/29; 55.17%) and the species *V. vermiformis* (11/29; 37.93%) being the most commonly identified. To a lesser extent, other FLA were observed (2/29; 6.89%), but no amoebae of the family Vahlkampfiidae were identified.

**Figure 4:**
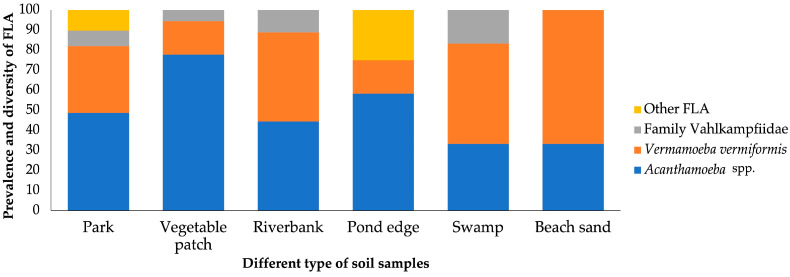
Distribution of free-living amoebae isolated in soil samples for types of soil.

According to soil type, all environments examined—parks, vegetable patches, riverbanks, pond edges, swamps, and beach sands—showed a 100% prevalence of environmental amoebic communities (Figure 4). However, the prevalence and diversity of environmental FLA varied significantly among these different soil types, reflecting the influence of environmental conditions on amoebic communities.

The park soils were characterised by a wide diversity comprising mostly the genus *Acanthamoeba* (19/39; 48.71%) followed by the species *V. vermiformis* (13/39; 33.33%). In addition, a notable presence of the members of the family Vahlkampfiidae was observed, with a representation close to 10%, while the other FLA contributed with a similar percentage.

Vegetable patch soils presented a microbial community dominated by *Acanthamoeba* spp., which constituted more than 60% of the total number of environmental FLA (14/18) identified in this ecosystem. *V. vermiformis* accounted for 16.67% (3/18), while the family Vahlkampfiidae was only observed in one sample.

The riverbank soils showed an equal diversity between *Acanthamoeba* spp. and *V. vermiformis* representing approximately 50%. *Vahlkampfia* sp. was also identified (1/9; 11.11%).

Soils located at the edge of the pond are notable for the presence of *Acanthamoeba* spp. *V. vermiformis* was present in a proportion of about 20%, while the other FLA represented about 25% of the total, indicating a higher diversity compared to other soil types. The family Vahlkampfiidae was not observed in this environment.

Swamp soils were characterised by an abundance of *V. vermiformis* (3/6; 50%) and *Acanthamoeba* spp. (2/6; 33.33%). In addition, *V. avara* was found in a proportion close to 20%, suggesting that the environmental conditions of the swamp, characterised by high humidity, accumulation of organic matter, and low oxygen levels, favour the coexistence of these amoebae.

Sand soils presented a microbial community dominated by *V. vermiformis* (2/3; 66.67%), although the genus *Acanthamoeba* was present in 33.33% (1/3). In contrast to other soil types, neither the family Vahlkampfiidae nor other FLA were present, suggesting a lower microbial diversity compared to other ecosystems.

Overall, of all the amoebae cultivated and monitored at 26 °C, *Acanthamoeba* spp. was the most frequently found amoeba in all soil types, followed by *V. vermiformis.* The presence of family Vahlkampfiidae and other FLA varied according to the environment, being more frequent in dry soils such as parks, vegetable patches, and other samples.

**Figure 5:**
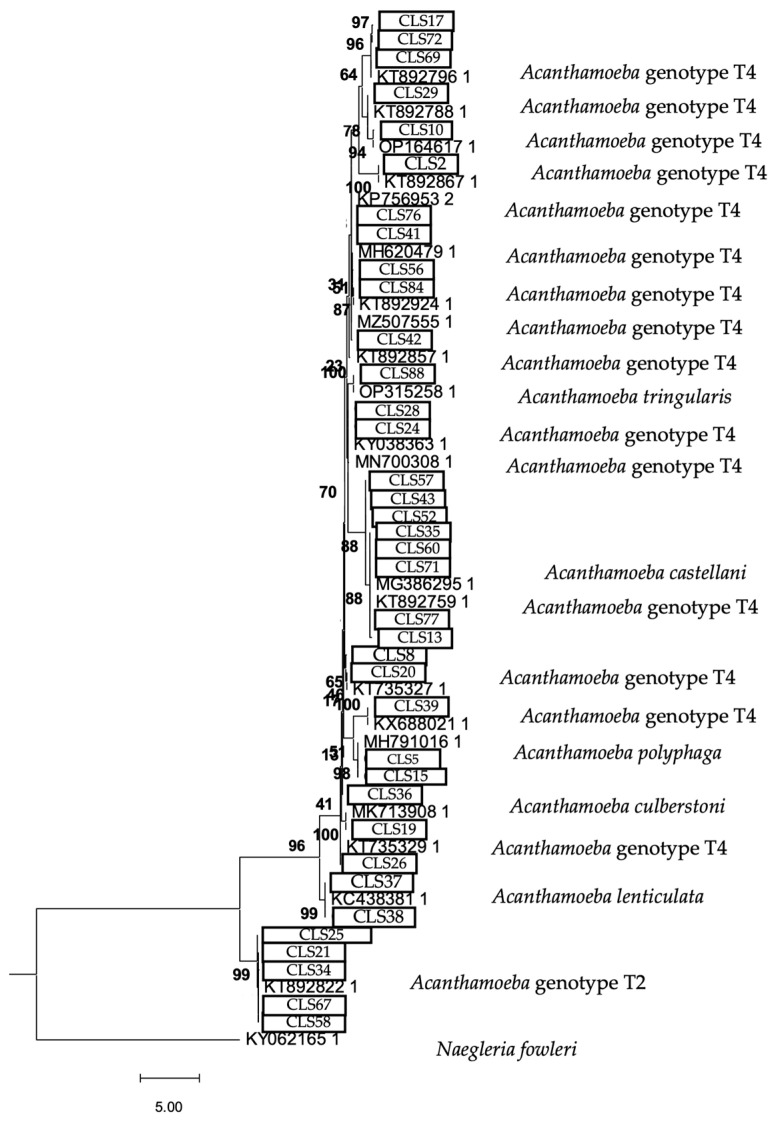
Maximum likelihood phylogenetic tree of the genus *Acanthamoeba* obtained based on the 18S rRNA gene analysis (1.311 nucleotide positions), showing the position of each isolate obtained in this study that is marked within the boxes. The tree is rooted with *Naegleria fowleri* as the outgroup. The percentage of replicate trees, in which the associated taxa are clustered together in the bootstrap test, is shown next to the branches (in bold). Scale bar = 5.00 substitutions/site.

**Figure 6:**
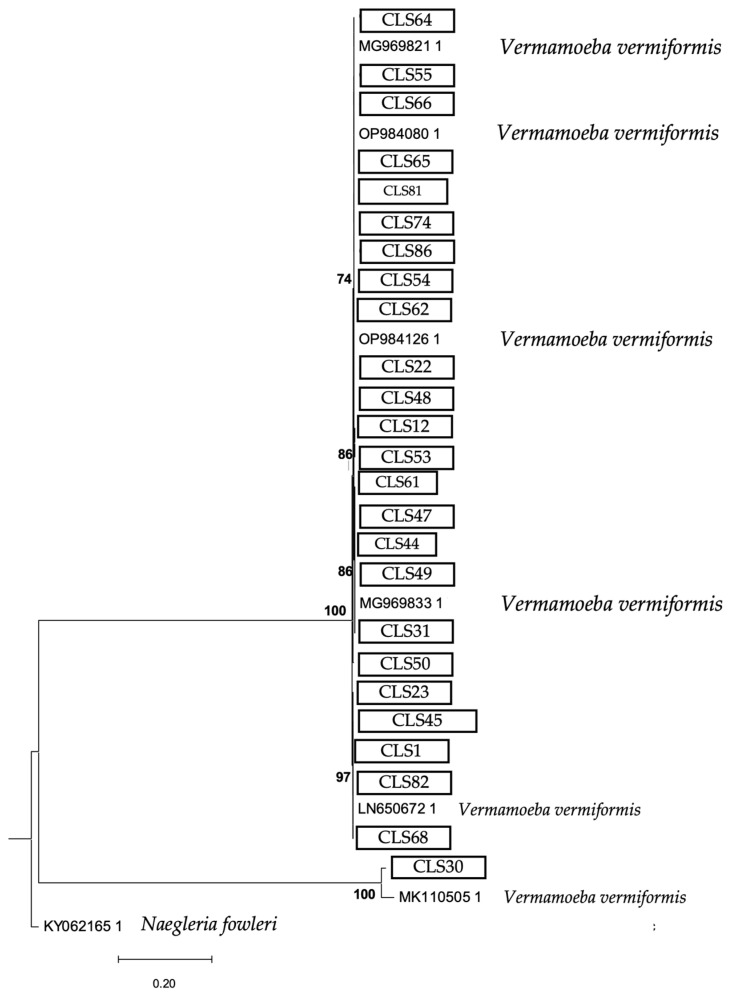
Maximum likelihood phylogenetic tree of the species *Vermamoeba vermiformis* obtained based on the 18S rRNA gene analysis (1.740 nucleotide positions) showing the position of each isolate obtained in this study that is marked within boxes. The tree is rooted with *Naegleria fowleri* as the outgroup. The percentage of replicate trees, in which the associated taxa are clustered together in the bootstrap test, is shown next to the branches (in bold). Scale bar = 0.20 substitutions/site.

**Figure 7:**
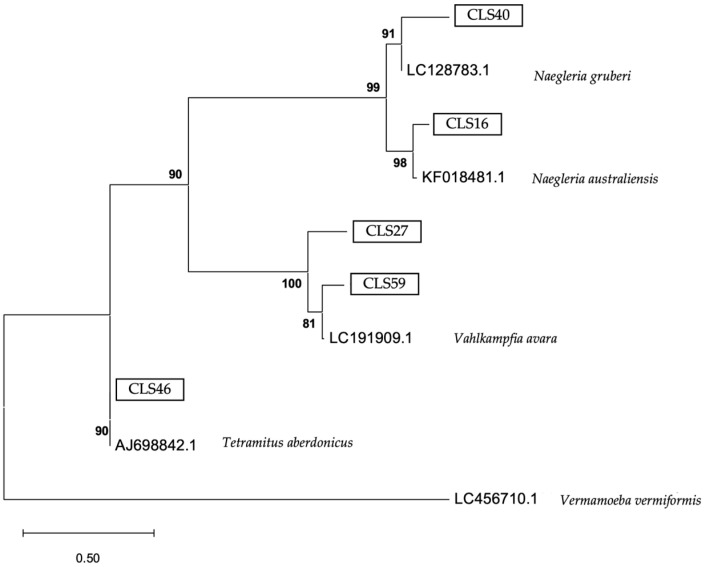
Maximum likelihood phylogenetic tree of the family Vahlkampfiidae obtained based on the internal transcribed sequences (ITS1 and ITS2 regions) and 5.8S sequence (625 nucleotide positions), showing the position of each isolate obtained in this study that is marked within boxes. The tree is rooted with *Vermamoeba vermiformis* as the outgroup. The percentage of replicate trees, in which the associated taxa are clustered together in the bootstrap test, is shown next to the branches (in bold). Scale bar = 0.50 substitutions/site.

The phylogenetic analysis of the 18S rRNA gene is shown in Figure 5, Figure 6 and Figure 7.

## 4. Discussion

The capacity of pathogenic FLA to cause serious illnesses in humans as well as animals has led to a major rise in research on these organisms in recent years [37]. Since FLA live in warm, humid conditions (mostly soil and freshwater), an elevation in the water’s surface temperature and floods brought on by heavy rainfall may increase the incidence of pathogenic FLA infections [38]. Considering pathogenic FLA are likely to infect their human host, it is essential to monitor their presence in soil, particularly species known to pose a threat to human health. To our knowledge, the present study is the first comprehensive long-term report of the occurrence of potentially pathogenic FLA in soil from Castilla y León. By combining culture methods with molecular analyses (conventional PCR and real-time PCR), we demonstrate that each province in Castilla y León exhibits distinct diversity and a prevalence of environmental FLA. Notably, for the first time, we document the presence of pathogenic and non-pathogenic FLA across various sampling efforts conducted over a full year, as detailed in the following discussion.

Therefore, the diversity and prevalence of environmental FLA in the soils of Castilla y León varied significantly according to the soil type, province, and sampling period. Considering the kind of soil analysed, in general, *Acanthamoeba* spp. predominated in samples taken from parks and vegetable patches, suggesting that these environments, rich in organic matter and nutrients, provide favourable conditions for their development. They also had a significant proportion of members of the family Vahlkampfiidae. On the other hand, *V. vermiformis* was isolated more frequently from riverbanks and pond edges, possibly due to the greater availability of moisture in these environments. Samples from beach sand and marshes showed a lower diversity of pathogenic FLA with *Acanthamoeba* spp. and *V. vermiformis*. These distribution patterns highlight the influence of soil type and the availability of environmental resources on the composition of microbial communities.

An analysis by province revealed that, in Salamanca, a dominance of *Acanthamoeba* spp. was observed in most soil types, with a balanced proportion of *V. vermiformis* in some periods, especially in parks and vegetable patches. In Valladolid, samples from the edges of ponds and parks showed a higher diversity of environmental FLA, including significant proportions of *V. vermiformis* and other FLA groups. In contrast, in Zamora, *Acanthamoeba* spp. predominated almost exclusively in parks and vegetable patches, while in riparian areas, members of the family Vahlkampfiidae were prominent in the second sampling. Finally, in Burgos, samples were characterised by low diversity, with *V. vermiformis* as the only group identified in riverbanks during the first two samplings and an exclusive presence of *Acanthamoeba* spp. in the third sampling.

In terms of sampling periods, a fluctuation in the diversity and abundance of environmental FLA was observed. In the first sampling, carried out between September and November, a diverse environmental amoebic community was observed, with the genus *Acanthamoeba* being the most abundant group, although other amoebae were present in a smaller number. This could be the case since this period in 2022 was reported as a warm time compared to other years [39]. The second sampling carried out between January and February reflected a small increase in diversity, with the presence of genera such as *Naegleria* and *Tetramitus*, probably due to higher levels of humidity derived from winter precipitation. Finally, the third sampling carried out between May and June, showed a more uniform distribution of *Acanthamoeba* spp. and *V. vermiformis*, although with less representation of other groups, which could reflect a balance between increasing temperature and decreasing soil moisture.

This variability observed throughout the three sampling periods can corroborate previous studies described by Bass and Bischoff [40], which show that the abundance and diversity of FLA in the environment depend on season, temperature, humidity, rainfall, pH, and nutrient availability. Furthermore, they have pointed out that amoeba diversity can increase during transitional seasons such as autumn due to optimal combinations of temperature and humidity. However, in winter, diversity tends to be lower as only species resistant to low temperatures or able to form cysts remain active. In contrast, our research showed a small increase in diversity, with new species of FLA being recorded due to increased humidity due to winter precipitation [41]. In fact, a study conducted at a tourist resort ‘Hierve el Agua’ in Oaxaca, Mexico, found a higher number of FLA species during the rainy season compared to the dry season [42].

On the other hand, according to the Köppen classification, the autonomous community of Castilla y León represents a continental Mediterranean climate with certain sub-climates distributed throughout each province. The provinces of Salamanca, Valladolid, and Zamora have a sub-climate (Csb) characterised by mild summers that offer a favourable balance for the diversity of environmental FLA during a large part of the year, as observed above. In contrast, Burgos, with a Dfb sub-climate, highlights a more stable abundance and diversity due to the continuous availability of humidity. This suggests that *V. vermifom* is species were mostly present in this province for this reason.

This investigation revealed a significant prevalence of the genus *Acanthamoeba*, with it being the most common across the four provinces evaluated. Its high frequency in the different soil types belonging to the selected provinces could be due to its cosmopolitan character, which includes a wide range of ecological niches shared with humans, as well as the high resistance of cysts, which can survive for several years in conditions of drought and low or high temperatures, among others [43]. The identification of the T4 genotype in multiple samples, especially in parks, vegetable patches, and other recreational areas located in densely populated provinces such as Salamanca and Valladolid, highlights its great importance from a public health perspective. This genotype is the most virulent, it is associated with AK, GAE, and epithelial lesions, and its presence in environments accessible to and frequented by the general population—including children, immunocompromised individuals, and contact lens wearers—could pose a potential risk. Furthermore, it is the most frequently identified genotype in human infection and environmental samples [44,45,46,47]. The molecular assays used in this research have also allowed us to identify the T2 genotype, which has been isolated worldwide from patients with AK and has been less frequently associated with GAE [48,49,50].

The finding of other FLA, such as *V. vermiformis*, as well as members of the family Vahlkampfiidae, suggests a rich microbial biodiversity in this region. Notably, the genera *Naegleria* and *Tetramitus*, isolated at specific periods, belong to the same family as *Naegleria fowleri*, the only species that is pathogenic to humans. This suggests that it may have shared the same niche as *N. fowleri.* The presence of *Naegleria australiensis* is remarkable, as research on this species in Spain is limited, with previous reports mainly from southern regions [51]. Furthermore, the identification of *Tetramitus aberdonicus* expands knowledge on the previously unreported ecology of these organisms in the terrestrial environments of the Iberian Peninsula.

Regarding the methodology, the use of qPCR for samples that tested negative by conventional PCR was likely motivated by its high sensitivity and ability to detect low levels of DNA, which may be missed by conventional PCR. qPCR has a lower limit of detection (LOD), allowing for the identification of amoebae even when their abundance is minimal or DNA is degraded. For instance, qPCR can detect as little as one amoeba per sample, making it particularly effective in cases where conventional PCR fails due to low template concentrations or inhibitory substances in the sample.

The phylogenetic analysis is a fundamental tool for understanding the diversity and evolutionary relationships of environmental FLA found in soil samples. In this study, the phylogenetic tree obtained using the maximum likelihood method confirmed the genotypic assignment of the obtained sequences, reinforcing the reliability of the results obtained by conventional PCR [52,53]. The *Acanthamoeba* sequences consistently clustered within the T4 genotype, known for its virulence and wide environmental distribution, underscoring the health relevance of its presence in urban and agricultural soils. On the other hand, the sequences corresponding to *V. vermiformis* formed a clearly defined clade, consistent with its phylogenetic profile described in the literature. This reinforces its role as a potential reservoir of bacterial pathogens, given its close relationship with strains previously implicated in opportunistic infections. Finally, the Vahlkampfiidae family tree showed a distinct phylogenetic grouping, revealing species such as *N. gruberi* and *T. aberdonicus*, among others, which contributes to the understanding of their diversity in terrestrial environments.

However, this study has potential biases or study limitations that need to be considered, as the incubation temperatures used limit the isolation of the environmental amoebic community to 26 °C, excluding species such as *N. fowleri* that thrive at higher temperatures, for example. On the other hand, a new methodology could be employed as metagenomic sequencing offers a comprehensive approach to identify amoebae and their associated microbiomes directly from environmental DNA. This method can detect unculturable or unknown species, extending the scope of diversity analysis beyond traditional techniques.

In addition to our findings, several studies have examined the presence of environmental FLA in soil across diverse geographical regions, highlighting their global distribution and ecological significance. For instance, research conducted in Bolivia, Israel, and Guadeloupe, among others, has documented the prevalence of the FLA community in soil samples, emphasising their adaptability to various environmental conditions [54,55]. Nevertheless, compared to studies conducted in the Canary Islands and other areas of Spain, where research has mainly focused on aquatic samples, this work provides a novel perspective by evaluating soil samples and highlighting the pathogenic potential of amoebae [19,56,57,58]. Similarly to the research of Magnet et al. [23], where the presence of *Acanthamoeba* in water treatment plants was explored, our findings highlight the need for surveillance in different environmental settings. The soils examined in this current study are soils with high human interaction, where people, and especially children, should be made aware of the danger in soil samples from parks and orchards, for example, which they come into contact with most frequently. Public health should be made aware of the human infections that can be encountered due to contact with soil [59].

## 5. Conclusions

This study represents the first comprehensive analysis of the presence and diversity of environmental FLA in the soils of the autonomous community of Castilla y León, with a predominance of *Acanthamoeba* spp. and *V. vermiformis*, reflecting a rich microbial biodiversity influenced by climatic and geographical factors. The findings underline the need for the continued surveillance and public awareness of potential risks when interacting with contaminated soils, promoting a better understanding and the prevention of emerging diseases related to these amoebae.

## Figures and Tables

**Table 1:** Locations of environmental soil samples analysed for FLA detection in the autonomous community of Castilla y León.

Sample	Locality	Province	Coordinates	Soil Type	Sampling Period
SS1	Salamanca	Salamanca	40.959703, −5.665465	Park	T1–T3
SS2	Salamanca	Salamanca	40.956167, −5.670507	Riverbank	T1–T3
SS3	Salamanca	Salamanca	40.9612710, −5.6556529	Park	T1–T3
SS4	Salamanca	Salamanca	40.967759, −5.657005	Park	T1–T3
SS5	Salamanca	Salamanca	40.967934, −5.651229	Park	T1–T3
SS6	Salamanca	Salamanca	40.966007, −5.680474	Park	T1–T3
SS7	Salamanca	Salamanca	40.964590, −5.682484	Park	T1–T3
SS8	Salamanca	Salamanca	40.962817, −5.677331	Park	T1–T3
SS9	Aldeatejada	Salamanca	40.946595, −5.681442	Edge of a pond	T1–T3
SS10	Galindo y Perahuy	Salamanca	40.925187, −5.805347	Vegetable patch	T1–T3
SS11	Trabanca	Salamanca	41.231362, −6.386124	Vegetable patch	T1–T3
SS12	Trabanca	Salamanca	41.231895, −6.383861	Vegetable patch	T1–T3
SS13	Espadaña	Salamanca	41.131510, −6.361370	Edge of a pond	T1–T3
SS14	Almendra	Salamanca	41.273762, −6.321856	Swamp	T1–T3
SS15	Almendra	Salamanca	41.270194, −6.334739	Swamp	T1–T3
SS16	Miranda de Azán	Salamanca	40.888489, −5.683101	Park	T1–T3
SS17	Miranda de Azán	Salamanca	40.874163, −5.683229	Edge of a pond	T1–T3
SS18	Miranda de Azán	Salamanca	40.875737, −5.683251	Vegetable patch	T1–T3
SS19	Miranda de Azán	Salamanca	40.875737, −5.683251	Vegetable patch	T1–T3
VS1	Valladolid	Valladolid	41.657199, −4.733434	Beach sand	T1–T3
VS2	Valladolid	Valladolid	41.579598, −4.661287	Edge of a pond	T1–T3
VS3	Valladolid	Valladolid	41.657347, −4.732622	Park	T1–T3
VS4	Valladolid	Valladolid	41.644965, −4.730745	Park	T1–T3
VS5	Valladolid	Valladolid	41.645413, −4.729856	Park	T1–T3
ZS1	Benavente	Zamora	42.003000, −5.677081	Park	T1–T3
ZS2	Benavente	Zamora	41.995946, −5.682736	Park	T1–T3
ZS3	Benavente	Zamora	41.996640, −5.676092	Vegetable patch	T1–T3
ZS4	Zamora	Zamora	41.495446, −5.754557	Riverbank	T1–T3
BS1	Burgos	Burgos	42.337932, −3.705546	Riverbank	T1–T3

## Data Availability

Data are contained within the article and supplementary materials.

## Supplementary Materials

The following supporting information can be downloaded at: https://www.mdpi.com/article/10.3390/microorganisms13051065/s1, Table S1: Additional details, results, including tables showing the presence of free-living amoebae in each of the soils from Castilla y León for three sampling.
