# Supplementary material for: Assessment of the Presence of Free-Living Amoebae in Soil Samples from the Northwest Region of Spain Using Culture and Molecular Assays
Source: Microorganisms. 2025 May 2;13(5):1065. doi: 10.3390/microorganisms13051065 (PMC12114036; doi:10.3390/microorganisms13051065)
Supplement: Supplementary file 1 [file microorganisms-13-01065-s001.zip › microorganisms-3572119-supplementary.pdf]

Table S1. FLA species isolated from the evaluated soil samples from the autonomous community of Castilla y León in different sampling (NNA: FLA growth in non-nutrient agar culture; PCR: FLA detection by PCR; homology (%) related to NCBI Database sequence).

Soil samples									
**Sampling**	**Sample code**	**Province**	**Soil type**	**NNA**	**PCR**	**q-PCR**	**Genus/species**	**Genbank code**	**Homology (%)**
T1	SS1	Salamanca	Park	+	-		Unidentified amoeba		
	SS2	Salamanca	Riverbank	+	+		*Acanthamoeba* sp. T4	CLS5	≥ 95%
	SS3	Salamanca	Park	+	-		Unidentified amoeba		
	SS4	Salamanca	Park	+	+		*Vahlkampfia* sp.	CLS7	≥ 95%
	SS5	Salamanca	Park	+	+		*Vermamoeba vermiformis*	CLS12	≥ 95%
	SS6	Salamanca	Park	+	+		*Vermamoeba vermiformis*	CLS1	≥ 95%
	SS7	Salamanca	Park	+	+		*Acanthamoeba* sp. T4	CLS2	≥ 95%
	SS8	Salamanca	Park	+	+		*Acanthamoeba* sp. T5	CLS3	≥ 95%
	SS9	Salamanca	Edge of a pond	+	+		*Acanthamoeba castellani* T4	CLS13	≥ 95%
	SS10	Salamanca	Vegetable patch	+	+		*Acanthamoeba* sp. T4	CLS28	≥ 95%
	SS11	Salamanca	Vegetable patch	+	+		*Vermamoeba vermiformis*	CLS23	≥ 95%
	SS12	Salamanca	Vegetable patch	+	+		*Acanthamoeba* sp. T4	CLS24	≥ 95%
	SS13	Salamanca	Edge of a pond	+	+		*Acanthamoeba palestinensis* T2	CLS25	≥ 95%
	SS14	Salamanca	Swamp	+	+		*Acanthamoeba* sp. T4	CLS26	≥ 95%
	SS15	Salamanca	Swamp	+	+		*Vahlkampfia* avara	CLS27	≥ 95%
	SS16	Salamanca	Park	+	+		*Acanthamoeba* sp. T4	CLS8	≥ 95%
	SS17	Salamanca	Edge of a pond	+	-		Unidentified amoeba		
	SS18	Salamanca	Vegetable patch	+	+		*Acanthamoeba* sp. T4	CLS10	≥ 95%
	SS19	Salamanca	Vegetable patch	+	-	+	*Acanthamoeba* sp. T4		
	VS1	Valladolid	Beach sand	+	+		*Vermamoeba vermiformis*	CLS14	≥ 95%
	VS2	Valladolid	Edge of a pond	+	+		*Acanthamoeba polyphaga* T4	CLS15	≥ 95%

	VS3	Valladolid	Park	+	+		*Naegleria australiensis*	CLS16	≥95%
	VS4	Valladolid	Park	+	+		*Acanthamoeba* sp. T4	CLS17	≥95%
	VS5	Valladolid	Park	+	-		Unidentified amoeba		
	ZS1	Zamora	Park	+	+		*Acanthamoeba culberstoni* T4B	CLS19	≥95%
	ZS2	Zamora	Park	+	+		*Acanthamoeba* sp. T4	CLS20	≥95%
	ZS3	Zamora	Vegetable patch	+	+		*Acanthamoeba palestinensis* T2	CLS21	≥95%
	ZS4	Zamora	Riverbank	+	+		*Vermamoeba vermiformis*	CLS22	≥95%
	BS1	Burgos	Riverbank	+	+		*Vermamoeba vermiformis*	CLS30	≥95%
T2	SS1	Salamanca	Park	+	+		*Vermamoeba vermiformis*	CLS31	≥95%
	SS2	Salamanca	Riverbank	+	-	+	*Acanthamoeba* sp. T4		
	SS3	Salamanca	Park	+	-	+	*Acanthamoeba* sp. T4		
	SS4	Salamanca	Park	+	+		*Acanthamoeba* sp. T2	CLS34	≥95%
	SS5	Salamanca	Park	+	+		*Acanthamoeba* sp. T4	CLS35	≥95%
	SS6	Salamanca	Park	+	+		*Acanthamoeba* sp. T4	CLS36	≥95%
	SS7	Salamanca	Park	+	+		*Acanthamoeba lenticulata* T5	CLS37	≥95%
	SS8	Salamanca	Park	+	+		*Acanthamoeba lenticulata* T5	CLS38	≥95%
	SS9	Salamanca	Edge of a pond	+	+		*Acanthamoeba* sp. T4	CLS39	≥95%
	SS10	Salamanca	Vegetable patch	+	+		*Naegleria gruberi*	CLS40	≥95%
	SS11	Salamanca	Vegetable patch	+	+		*Acanthamoeba* sp. T4	CLS41	≥95%
	SS12	Salamanca	Vegetable patch	+	+		*Acanthamoeba* sp. T4	CLS42	≥95%
	SS13	Salamanca	Edge of a pond	+	+		*Acanthamoeba* sp. T4	CLS43	≥95%
	SS14	Salamanca	Swamp	+	+		*Vermamoeba vermiformis*	CLS44	≥95%
	SS15	Salamanca	Swamp	+	+		*Vermamoeba vermiformis*	CLS45	≥95%
	SS16	Salamanca	Park	+	+		*Tetramitus aberdonicus*	CLS46	≥95%

	SS17	Salamanca	Edge of a pond	+	+		*Vermamoeba vermiformis*	CLS47	≥95%
	SS18	Salamanca	Vegetable patch	+	+		*Vermamoeba vermiformis*	CLS48	≥95%
	SS19	Salamanca	Vegetable patch	+	+		*Vermamoeba vermiformis*	CLS49	≥95%
	VS1	Valladolid	Beach sand	+	+		*Vermamoeba vermiformis*	CLS50	≥95%
	VS2	Valladolid	Edge of a pond	+	-		Unidentified amoeba		
	VS3	Valladolid	Park	+	+		*Acanthamoeba* sp. T4	CLS52	≥95%
	VS4	Valladolid	Park	+	+		*Vermamoeba vermiformis*	CLS53	≥95%
	VS5	Valladolid	Park	+	+		*Vermamoeba vermiformis*	CLS54	≥95%
	ZS1	Zamora	Park	+	+		*Acanthamoeba* sp. T4	CLS56	≥95%
	ZS2	Zamora	Park	+	+		*Acanthamoeba* sp. T4	CLS57	≥95%
	ZS3	Zamora	Vegetable patch	+	+		*Acanthamoeba palestinensis* T2	CLS58	≥95%
	ZS4	Zamora	Riverbank	+	+		*Vahlkampfia* sp.	CLS59	≥95%
	BS1	Burgos	Riverbank	+	+		*Vermamoeba vermiformis*	CLS55	≥95%
T3	SS1	Salamanca	Park	+	+		*Acanthamoeba castellani* T4	CLS60	
	SS2	Salamanca	Riverbank	+	+		*Vermamoeba vermiformis*	CLS61	≥95%
	SS3	Salamanca	Park	+	+		*Vermamoeba vermiformis*	CLS62	≥95%
	SS4	Salamanca	Park	+	+		*Vermamoeba vermiformis*	CLS63	≥95%
	SS5	Salamanca	Park	+	+		*Vermamoeba vermiformis*	CLS64	≥95%
	SS6	Salamanca	Park	+	+		*Vermamoeba vermiformis*	CLS65	≥95%
	SS7	Salamanca	Park	+	+		*Vermamoeba vermiformis*	CLS66	≥95%
	SS8	Salamanca	Park	+	+		*Acanthamoeba* sp. T2	CLS67	≥95%
	SS9	Salamanca	Edge of a pond	+	+		*Vermamoeba vermiformis*	CLS68	≥95%
	SS10	Salamanca	Vegetable patch	+	-+		*Acanthamoeba* sp. T4	CLS69	≥95%
	SS11	Salamanca	Vegetable patch	+	+		*Acanthamoeba* sp. T4	CLS70	≥95%
	SS12	Salamanca	Vegetable patch	+	+		*Acanthamoeba castellani* T4	CLS71	≥95%

	SS13	Salamanca	Edge of a pond	+	+		*Acanthamoeba* sp. T4	CLS72	≥95%
	SS14	Salamanca	Swamp	+	+		*Acanthamoeba quina* T4	CLS73	≥95%
	SS15	Salamanca	Swamp	+	+		*Vermamoeba vermiformis*	CLS74	≥95%
	SS16	Salamanca	Park	+	+	+	*Acanthamoeba* sp. T4		
	SS17	Salamanca	Edge of a pond	+	+		*Acanthamoeba* sp. T4	CLS76	≥95%
	SS18	Salamanca	Vegetable patch	+	+		*Acanthamoeba* sp. T4	CLS77	≥95%
	SS19	Salamanca	Vegetable patch	+	+	+	*Acanthamoeba* sp. T4		
	VS1	Valladolid	Beach sand	+	+		*Acanthamoeba* sp. T4	CLS79	≥95%
	VS2	Valladolid	Edge of a pond	+	-		Unidentified amoeba		
	VS3	Valladolid	Park	+	+		*Vermamoeba vermiformis*	CLS81	≥95%
	VS4	Valladolid	Park	+	+		*Vermamoeba vermiformis*	CLS82	≥95%
	VS5	Valladolid	Park	+	-		Unidentified amoeba		
	ZS1	Zamora	Park	+	-	+	*Acanthamoeba* sp. T4		
	ZS2	Zamora	Park	+	+		*Vermamoeba vermiformis*	CLS86	≥95%
	ZS3	Zamora	Park	+	-	+	*Acanthamoeba* sp. T4		≥95%
	ZS4	Zamora	Vegetable patch	+	-		*Acanthamoeba tringularis* T4	CLS88	
	BS1	Burgos	Riverbank	+	+		*Acanthamoeba* sp. T4	CLS84	≥95%
